# A harmonized individual-level phytoplankton trait dataset from Mediterranean and Black Sea transitional waters

**DOI:** 10.3897/BDJ.14.e196603

**Published:** 2026-10-12

**Authors:** Jessica Titocci, Maira Laraib, Teodoro Semeraro, Alberto Basset

**Affiliations:** 1 Italian National Research Council (CNR), Institute for Research on Terrestrial Ecosystems (IRET), Lecce, Italy Italian National Research Council (CNR), Institute for Research on Terrestrial Ecosystems (IRET) Lecce Italy; 2 University of Salento, Department of Biological and Environmental Sciences and Technologies (DiSTeBA), Lecce, Italy University of Salento, Department of Biological and Environmental Sciences and Technologies (DiSTeBA) Lecce Italy; 3 National Biodiversity Future Centre (NBFC), Palermo, Italy National Biodiversity Future Centre (NBFC) Palermo Italy; 4 LifeWatch ERIC Service Centre, Campus Ecotekne, Lecce, Italy LifeWatch ERIC Service Centre, Campus Ecotekne Lecce Italy

**Keywords:** biodiversity, coastal lagoons, phytoplankton, morphological traits, cell size, shape, FAIR data

## Abstract

**Background:**

Transitional waters are dynamic coastal ecosystems located at the interface between freshwater and marine environments. They are characterized by pronounced environmental variability resulting from the interaction between geomorphology, freshwater inputs, tidal exchanges, climatic variability, and human activities. These factors generate high habitat heterogeneity and complex ecological dynamics.

Phytoplankton communities inhabiting transitional waters are particularly sensitive to both natural gradients and anthropogenic pressures, often exhibiting marked spatial and seasonal patterns in species composition, abundance and functional structure. In response to fluctuating environmental conditions, phytoplankton taxa display a wide range of morphometric and functional adaptations, making trait-based approaches especially suitable for investigating community organization and ecosystem functioning in these ecosystems.

Although a large amount of phytoplankton trait data is available, its use could be enhanced through improved data harmonization and interoperability. The availability of FAIR (Findable, Accessible, Interoperable and Reusable) phytoplankton trait data from transitional waters, coupled with associated environmental information, is therefore essential to support comparative analyses and to improve our understanding of trait–environment relationships in these vulnerable ecosystems.

**New information:**

In this study, we present two complementary and FAIR datasets derived from coordinated monitoring activities conducted in transitional water ecosystems across the Mediterranean and Black Sea regions. The phytoplankton dataset comprises individual-level morpho-functional trait measurements collected at 14 sites, including 104,687 records from 242 taxa belonging to 10 phyla. Recorded traits include cell size, shape, density, biovolume and estimated carbon content and biomass values, enabling analyses at multiple levels of biological organization, from individuals to functional guilds and whole communities. The abiotic dataset includes in situ measurements of key physicochemical variables describing water quality conditions, such as salinity, conductivity, temperature, pH, dissolved oxygen, oxygen saturation and total dissolved solids. Nutrient concentrations are also provided, including nitrite (NO₂), nitrate (NO₃), ammonium (NH₄), dissolved inorganic nitrogen, phosphate (PO₄) and silicate (SiO₂). In addition, the dataset also includes information, when available, on the main physiographic and hydrological characteristics of the studied transitional waters, including water depth, tidal range, inlet morphology (outlet length and width), sinuosity index, surface area and hydrological connectivity. Qualitative descriptors of anthropogenic pressure and protection status are also included, such as the presence of Ramsar designation, Natura 2000 sites, local protection plans and indicators of human disturbance. Together, these variables characterize microtidal, nutrient-rich lagoon ecosystems that differ in their environmental conditions and degrees of anthropogenic impact, providing a comprehensive environmental context for trait-based analyses of phytoplankton communities.

Both datasets have been harmonized to ensure semantic consistency, interoperability and reusability. They are accompanied by comprehensive metadata and are openly accessible through the LifeWatch Italy Data Portal and Metadata Catalogue (doi: https://doi.org/10.48372/x9qx-4t72). Data quality and usability have been validated through their application in previously published studies focusing on morpho-functional analyses, phytoplankton guild classification and hypothesis testing across different levels of data aggregation. Together, these FAIR-compliant datasets represent a robust and reusable resource for advancing trait-based research and functional ecology in dynamic transitional water ecosystems.

## Introduction

Over the last few decades, trait-based approaches have become a central framework in phytoplankton community ecology, providing powerful tools to investigate patterns of biodiversity, community assembly, and ecosystem functioning (Litchman and Klausmeier 2008, Litchman et al. 2010, Litchman et al. 2015, Weithoff and Beisner 2019, Martini et al. 2021). By focusing on measurable organismal characteristics that influence ecological performance, these approaches enable researchers to link species composition and their roles with environmental conditions and ecosystem processes (McGill et al. 2006, Violle et al. 2007).

Phytoplankton represent an ideal model system for trait-based ecological studies. This diverse group of microscopic photosynthetic organisms accounts for approximately 40% of global primary production and plays a key role in aquatic biogeochemical cycles (Falkowski 1994, Litchman et al. 2015). Phytoplankton communities influence climate regulation (Chien et al. 2023), water quality (Carvalho et al. 2012, Vadrucci et al. 2013), and trophic dynamics in aquatic ecosystems (Franzè et al. 2022). Due to their rapid growth rates and sensitivity to environmental variability and anthropogenic pressures, they are widely used as indicators of ecological status and ecosystem change (Beaugrand 2005, Padisák et al. 2006, Ptacnik et al. 2009, Racault et al. 2014). The relatively simple morphology of phytoplankton cells, combined with well-defined functional traits, makes phytoplankton communities particularly suitable for trait-based analyses (Litchman and Klausmeier 2008, Violle et al. 2007, Laraib et al. 2023, Titocci and Fink 2023).

Functional and morphometric traits strongly influence phytoplankton ecological strategies, affecting individual fitness, resource acquisition, and competitive ability, with cascading effects at population, species, and community levels (Fontana et al. 2015, Hamer et al. 2022). Individual-level trait measurements are particularly valuable because they allow the investigation of intra-specific variability and fine-scale ecological responses to environmental drivers (Webb et al. 2010, Weithoff and Beisner 2019). However, despite the growing importance of trait-based approaches, phytoplankton trait datasets with individual-level resolution remain relatively scarce. Most available databases report trait values aggregated at the species level and primarily focus on marine or freshwater ecosystems (Davies et al. 2016, Deemer et al. 2023, Laplace-Treyture et al. 2021). As a result, harmonized trait data that allow the investigation of intra-specific variability are still limited, particularly for transitional water ecosystems.

These environments represent highly dynamic interfaces between freshwater and marine systems and are characterized by strong environmental gradients, high spatial heterogeneity, and multiple anthropogenic pressures (Sangiorgio et al. 2008, Basset et al. 2013). Furthermore, phytoplankton trait data remain scarce in specific regions, such as Mediterranean and Black Sea coastal lagoons, highlighting the need for FAIR datasets to support functional diversity research in transitional waters. Improving the availability of harmonized FAIR phytoplankton trait data from transitional waters can support comparative analyses, trait–environment studies, and investigations of phytoplankton community organization in these vulnerable ecosystems.

Here, we present a harmonized individual-level phytoplankton trait dataset comprising 104,687 records from 248 taxa collected across 14 transitional water sites in Mediterranean and Black Sea coastal lagoons, located in Albania, Bulgaria, Greece, Italy, and Romania, spanning a range of environmental gradients and levels of anthropogenic pressure. The phytoplankton dataset includes morpho-functional traits derived from microscopic observations and image analysis, such as cell size, shape, and structural characteristics, providing detailed information on phytoplankton species composition, ecological interactions, and functional diversity.

The study sites represent microtidal and non-tidal, nutrient-rich depositional ecosystems that differ in physicochemical conditions, hydrological features, and degrees of human impact. The dataset therefore provides a comprehensive characterization of the morphological structure of phytoplankton communities across multiple transitional water systems. Importantly, trait measurements were conducted at the individual level, enabling high-resolution analyses of intra-specific variability and population-level responses to environmental gradients.

The dataset has been fully harmonized to ensure interoperability and reusability and is openly accessible through the LifeWatch Italy Data Portal, the Italian National Distributed Node of the LifeWatch ERIC research infrastructure (https://doi.org/10.48372/x9qx-4t72). By providing standardized individual-level morpho-functional trait data, this resource contributes to improving the availability of FAIR phytoplankton trait information and supports future trait-based and comparative ecological studies in coastal transitional ecosystems.

## General description

### Purpose

This dataset contains individual-level morpho-functional trait records of phytoplankton communities collected from multiple lagoon sites across Mediterranean and Black Sea transitional waters. It extends the existing inventory of phytoplankton trait data for transitional aquatic ecosystems and complements available marine and freshwater trait-based data resources.

The dataset enables a wide range of analyses, including the assessment of intra- and interspecific trait variability, functional diversity patterns and community structure along environmental gradients. By providing standardized individual-level measurements, it supports fine-scale investigations of phytoplankton community dynamics and trait–environment relationships.

The dataset has also been used as a useful case within the Phytoplankton Virtual Research Environment (https://phytovre.itineris.cnr.it/), where it contributed to data harmonization, trait-based integration and community-level analyses, further demonstrating its applicability and reusability.

## Project description

### Title

INTERREG IIIB CADSES TWReference Net Project. Water bodies in Europe: Integrative Systems to assess Ecological status and Recovery, funded by the European Union under the 7^th^ Framework Programme, Theme 6 (Environment including Climate Change).

### Personnel

The project was coordinated by Prof. Alberto Basset, with the contribution of multiple experts involved in field campaigns, sampling and data collection across study sites in Albania, Bulgaria, Greece, Italy and Romania.

### Study area description

The dataset includes phytoplankton and environmental data collected from transitional water ecosystems located in the northern Mediterranean and western Black Sea regions (Fig. 1). Mediterranean sites comprised Alimini, Cesine, Torre Guaceto, Margherita di Savoia, Grado Valli da Pesca, Grado Marano (Italy, sites 1-6), Karavasta, Narta and Patok (Albania, sites 7-9); and Agiasma and Logarou (Greece, sites 10-11). Western Black Sea sites included Varna bay (Bulgaria, site 11) and Sinoe lagoon and Lehaova (Romania, sites 13-14). The study sites are heterogeneous in terms of their physicochemical conditions and hydro-morphological features, as well as with respect to natural variability and anthropogenic pressures acting at both water body and catchment scales. This heterogeneity provides a broad environmental context for the investigation of phytoplankton morpho-functional trait variability in transitional water ecosystems.

### Design description

Data were collected during two sampling campaigns conducted in autumn 2004 (October) and spring 2005 (April) within the main habitat types of each lagoon. A hierarchical sampling design was adopted to capture both seasonal and spatial variability in phytoplankton communities (Vadrucci et al. 2008). Water samples were collected from sampling stations distributed across five regions and 14 sites. The number of sampling stations per site ranged from one to seven: Agiasma (4), Alimini (2), Cesine (3), Grado Marano (3 in October and 4 in April), Grado Valli da Pesca (4), Karavasta (2), Lehaova (1 in October and 2 in April), Logarou (7), Margherita di Savoia (4 in October and 5 in April), Narta (3), Patok (2), Sinoe (3), Torre Guaceto (2), and Varna (5). At each sampling station, approximately two to four replicate water samples were collected.

### Funding

This study was carried out within the framework of the INTERREG IIIB CADSES TWReference Net Project, with financial support from the Italian Ministry of University and Research (MIUR) and the University of Salento (UPB RIAF.RIAF1 – Planning and Budget Office, expense item 10211009). The dataset was subsequently provided for research activities within LifeWatchPLUS (CIR01_00028). Data processing, harmonization and FAIRification together with the preparation of this publication were funded by EU – Next Generation EU, Mission 4 “Education and Research”, Component 2 “From research to business”, Investment 3.1 “Fund for the realisation of an integrated system of research and innovation infrastructures”, under the project IR0000032 – ITINERIS (Italian Integrated Environmental Research Infrastructures System), CUP B53C22002150006.

## Sampling methods

### Study extent

The dataset comprises individual-level morpho-functional trait data of phytoplankton communities collected from 14 coastal lagoon sites located in Mediterranean and Black Sea transitional waters. The study sites are distributed across five countries: Albania, Bulgaria, Greece, Italy and Romania, covering a broad geographical and environmental gradient representative of microtidal and non-tidal lagoon ecosystems. Geographic coordinates for each sampling site are provided in the dataset metadata, allowing precise spatial referencing and geospatial analyses.

### Sampling description

Sampling was conducted during two seasonal campaigns, in autumn (October 2004) and spring (April 2005). Within each lagoon, two to three heterogeneous areas were selected based on habitat type classification to capture spatial variability in phytoplankton communities. Water samples were collected at a depth of 0.5 m using a Ruttner bottle. Samples were transferred to PET bottles, preserved with 1% Lugol’s iodine solution (15 ml L⁻¹), stored in darkness at 4 °C, and analyzed within two months of collection.

Abiotic parameters of the water column, including temperature, salinity, pH and dissolved oxygen, were measured in situ at each sampling station using a multiparametric probe (WTW Multi 3500i). Additional water samples were collected for macronutrient and processed immediately upon arrival at the laboratory following standard methods described by Strickland and Parsons 1972.

### Quality control

During data collection, a standardized sampling protocol was applied across all sampling locations to ensure data consistency and replicability. All samples were collected, identified, and measured by a team of qualified researchers and taxonomists.

Data curation and validation procedures were implemented to ensure the accuracy and consistency of both data and metadata. Phytoplankton and abiotic records were organized using the Data Template Builder tool (https://phytovre.itineris.cnr.it/data-template-builder/) of the Phytoplankton Virtual Research Environment (https://phytovre.itineris.cnr.it). This tool supports the generation of standardized and semantically interoperable data templates by assigning descriptive labels and harmonizing parameter names. The template incorporated concepts from the Darwin Core standards (Wieczorek et al. 2012), the Traits Thesaurus (Titocci et al. 2024), and other controlled vocabularies including MarineRegions (Marine Regions 2024), EnvThes (Grandin et al. 2013), Sandy beach Thesaurus (Onen-Tarantini et al. 2025), Phytoplankton VRE Thesaurus (Titocci and Semeraro 2026), NERC vocabulary (https://vocab.nerc.ac.uk/) to generate structured data tables for both phytoplankton and abiotic datasets (Table 1 and 2).

Taxonomic classification and validation were performed using the AlgaeBase repository (Guiry 2013). Both the original taxonomic name and the validated name are provided in the dataset through the Darwin Core data properties *verbatimIdentification* and *acceptedNameUsage*, respectively. This approach preserves the original identification while ensuring taxonomic standardization and traceability.

Morphological trait (shape) associations and linear dimension measurements were validated using the Atlas of Shapes web service (https://phytovre.itineris.cnr.it/vre/shapes-groups/) available within the Phyto VRE.

The datasets were also used for the development, testing, and validation of an analytical workflow (https://phytovre.itineris.cnr.it/workflow-5/) within the Phyto VRE designed to investigate spatial and temporal patterns in phytoplankton community structure by combining univariate and multivariate statistical approaches. It further supported the development of a dedicated case study (https://phytovre.itineris.cnr.it/use-case-wf-5/) within the Phyto VRE, where it served as a reference dataset for data harmonization, trait-based integration, and community-level analyses.

Following validation and curation, the dataset was archived and preserved in the LifeWatch Italy data portal. The data were formatted as column-oriented tables in comma-separated values (.csv) format. Metadata were provided using the Ecological Metadata Language (EML 2.2.0) standard in extensible markup language (.xml) format to ensure long-term usability and interoperability.

Finally, the data include information that has been previously published in peer-reviewed scientific journals (Sabetta et al. 2008, Vadrucci et al. 2008, Vadrucci et al. 2013).

### Step description

Phytoplankton subsamples were analyzed under an inverted microscope (Nikon T300E) at 400× magnification following the Utermöhl method (Utermöhl 1958). For each sample, when possible, a minimum of 400 cells were counted, measured, and identified to ensure statistically robust estimates of cell size and taxonomic composition. Taxonomic identification was performed using specific monographs, manuals, and phytoplankton atlases (Al-Kandari et al. 2009, Haraguchi and Odebrecht 2010, Pey et al. 2014, Sournia 1986, Tomas 1997). All cells were identified to the lowest possible taxonomic level. When species-level identification was not possible, qualifiers such as sp., spp., and cf. were used to indicate the closest assignable taxonomic rank. Geometric shapes were attributed based on cell morphology, including colonial, coenobial, and filamentous species in cases where individual cells were not directly observable (Stanca et al. 2012). Cell length and width were recorded together with additional linear dimensions specific to each geometric shape. These additional measurements were coded using alphabetical identifiers (e.g., length as “a” or “l”; width as “b”, “d”, or “h”). Morphological information were recorded in the dataset through the field shape, together with additional details on measurements and diagnostic features provided in measurementRemarks and taxonRemark, including information on the presence of internal and external cellular structures (e.g., theca, flagella).

After taxonomic identification, the biovolume of each cell was calculated according to its associated geometric shape using the equations and visual references provided by the Phytoplankton “Atlas of Shapes” web service. Trait computation was performed through the dedicated “Trait Computation” e-service implemented in Python within the Phyto VRE. This service computes cell density (cells L⁻¹) and automatically assigns the appropriate geometric formula based on taxon name and cell shape to calculate biovolume (µm³). Cell biovolume was subsequently converted into individual cellular carbon content (pg C cell⁻¹) following the equations proposed by Menden-Deuer and Lessard 2000. Biomass values (ng) were derived from biovolume using a conversion factor of 1.03. Density data were not available for the Grado Marano and Grado Valli da Pesca lagoons; therefore, these systems were excluded from quantitative analyses based on density.

## Geographic coverage

### Description

Phytoplankton and abiotic data were collected from 14 transitional water ecosystems located in Mediterranean and Black Sea coastal lagoons. The Northern Mediterranean sites comprise Karavasta, Narta, Patok, Alimini, Cesine, Grado Marano, Grado Valli da Pesca, Margherita di Savoia, Torre Guaceto, Agiasma, and Logarou, situated in Albania, Italy, and Greece. The Western Black Sea sites include Sinoe, Lehaova, and Varna, located in Romania and Bulgaria.

### Coordinates

34.7052260662 and 47.0818731873 Latitude; 10.7640205282 and 29.7132398146 Longitude.

## Taxonomic coverage

### Description

The dataset comprises 104,687 phytoplankton records assigned to 242 accepted taxa, distributed across 10 phyla, 22 classes, 67 orders, 100 families, and 136 genera. Among the 242 accepted taxa, 62.4% (151 taxa) were identified at the species level. Based on total density, Chlorophyta was the most represented phylum in the dataset (37.39%), followed by Cyanobacteria (23.58%) and Cryptista (15.12%) (Fig. 2a). In contrast, when considering total biomass, Dinoflagellata represented the largest contribution (36.29%), followed by Heterokontophyta (20.08%), Cyanobacteria (18.92%), and Chlorophyta (17.23%) (Fig. 2b). This difference reflects the larger cell size typically observed in dinoflagellates and diatoms compared with smaller phytoplankton groups.

In terms of taxonomic richness, Heterokontophyta was the most diverse group, with 92 taxa (*data not shown*), followed by Dinoflagellata (69 taxa) and Chlorophyta (39 taxa). Cyanobacteria included 17 taxa, Haptophyta 9, Cryptista 6, Euglenophyta 5, and Charophyta 3, while Bacillariophyt and Ciliophora were each represented by a single taxon (*Melosira* sp. and *Mesodinium rubrum*). The single representative of Ciliophora was the ciliate *Mesodinium rubrum*, which has been recognized as part of the photosynthetic plankton community (Lips and Lips 2017). However, since only cell measurements but no density data were available for *Melosira* sp. and *Mesodinium rubrum*, these taxa were excluded from the analyses. Undetermined records, including Phytoflagellates, Microflagellates, and Phytoplankton undetermined, were grouped into an “Others” category for compositional analyses, but were excluded from taxonomic richness and Shannon diversity calculations. This category accounted for 19.96% of the records and 4.98% of the total density. These records mainly include cells exhibiting flagella and chloroplasts that could not be reliably identified at the species level or assigned to higher taxonomic ranks. Phytoplankton community structure showed noticeable differences between Mediterranean and Black Sea lagoons (Fig. 3). Mediterranean lagoons generally exhibited a more heterogeneous community composition, with alternating dominance of Cryptista, Cyanobacteria and Chlorophyta in terms of density. In contrast, several Black Sea lagoons, particularly Sinoe and Varna, showed a stronger contribution of Cyanobacteria and Dinoflagellata, especially when biomass was considered. Taxonomic richness also differed among lagoons and regions. Mediterranean lagoons displayed richness values typically ranging between 23 and 49 taxa, whereas Black Sea lagoons showed higher values, ranging from 46 to 79 taxa, with the highest richness observed in Varna. Shannon diversity followed a similar pattern. Mean values based on density ranged from 0.56 to 2.56 across lagoons, while biomass-based values ranged from 1.11 to 2.10, indicating differences in community evenness among lagoons. Overall, these results indicate that Mediterranean lagoons tend to host phytoplankton communities in which taxa are more evenly distributed, whereas Black Sea lagoons often show stronger dominance by specific functional groups, particularly cyanobacteria or dinoflagellates.

## Traits coverage

Cell size and shape represent key functional traits influencing phytoplankton ecology, physiology, and evolutionary strategies (Finkel et al. 2007). In the dataset, morphological traits were described using cell geometry and associated metrics including biovolume, carbon content, and biomass.

Overall, 24 different phytoplankton cell shapes were identified in the dataset, although only 23 could be included in the density-based analysis, as density values were unavailable for *Climacosphenia* sp. (prism on elliptic base + parallelepiped, one record). When considering the distribution of shapes in terms of total phytoplankton density (Fig. 4), the most dominant morphologies were cube-shaped cells (19.3%) and cylindrical cells (19.2%), which together accounted for nearly 40% of the total density. These were followed by cone + half sphere (15.2%), prolate spheroid (10.8%), and sphere (10.0%). Additional relevant morphologies included cylinder + 2 cones (8.6%) and prism on elliptic base (8.5%), while the remaining shapes individually contributed less than 3% of the total phytoplankton density. For functional interpretation, cell shapes were further grouped into simple and complex morphological categories according to their geometric structure. Simple shapes included geometrically regular forms such as spheres, cylinders, cubes, ellipsoids, and related variants, whereas complex shapes consisted of combinations of multiple geometric shapes (e.g., cone + half sphere, cylinder + 2 cones, and other composite structures). Simple shapes accounted for approximately 72% of the total phytoplankton density, while complex shapes contributed 28% (Fig. 4). This pattern indicates that although complex morphologies can contribute significantly to phytoplankton density, simple geometric shapes dominate the shape structure of the community. When comparing the two geographical regions, no pattern in shape richness comparable to that observed for species richness was evident *(data not shown)*. Mediterranean sites contained 11–14 cell shapes per site and Black Sea sites 14–16, with 21 and 19 shapes recorded across the two regions overall, respectively, of which 16 were shared. Differences were more apparent in shape composition: cone + half sphere showed the highest relative contribution to cell density in Mediterranean sites (43.8%), whereas cylindrical (29.8%) and cube-shaped cells (18.5%) predominated in Black Sea sites. Cylindrical forms were mainly associated with *Binuclearia lauterbornii*, *Cyclotella caspia*, *Anabaena*, and *Planktolyngbya* spp., while cube-shaped cells were predominantly represented by *Merismopedia* and *Crucigenia*. Simple shapes also showed a higher mean relative contribution to cell density in Black Sea sites (78.0%) than in Mediterranean sites (50.8%), where the contribution of simple and complex shapes was more balanced and more variable among localities.

## Usage licence

### Usage licence

Other

### IP rights notes

This work, the dataset and associated metadata are licensed under the Creative Commons (CC BY 4.0).

## Data resources

### Data package title

Individual level trait-based dataset of phytoplankton communities from the coastal lagoons of the Mediterranean and Black Sea

### Resource link

https://doi.org/10.48372/x9qx-4t72

### Alternative identifiers

https://data.lifewatchitaly.eu/handle/123456789/326359.3

### Number of data sets

2

### Data set 1.

#### Data set name

PhytoplanktonTraits_Mediterranean_BlackSea_CoastalLagoons_v1.2.1.csv

#### Data format

UTF-8 .csv (comma_separated values format).

#### Download URL

https://data.lifewatchitaly.eu/bitstreams/bef08f92-1c68-491a-b9bb-9430f55667d9/download

#### Data format version

1.2

#### Description

The phytoplankton dataset includes detailed records of species occurrence, cell density, geometric cell shapes, biovolume, carbon content and biomass estimates. The dataset captures spatial and seasonal variability in phytoplankton community composition, phytoplankton traits and biomass under contrasting ecological conditions.

Table 1. Phytoplankton data table with the terminologies used as column headers, their definitions and URIs (Uniform Resource Identifiers), based on the adopted controlled vocabularies used.

**Data set 1.** 

Column label	Column description
catalogNumber	An identifier (preferably unique) for the record within the data set or collection. http://rs.tdwg.org/dwc/terms/catalogNumber
organismQuantity	A number or enumeration value for the quantity of dwc:Organisms. http://rs.tdwg.org/dwc/terms/organismQuantity
organismQuantityType	The type of quantification system used for the quantity of dwc:Organisms. http://rs.tdwg.org/dwc/terms/organismQuantityType
eventID	An identifier for the set of information associated with a dwc:Event (something that occurs at a place and time). May be a global unique identifier or an identifier specific to the data set. http://rs.tdwg.org/dwc/terms/eventID
parentEventID	An identifier for the broader dwc:Event that groups this and potentially other dwc:Events. http://rs.tdwg.org/dwc/terms/parentEventID
country	The name of the country or major administrative unit in which the dcterms:Location occurs. http://rs.tdwg.org/dwc/terms/country
countryCode	The standard code for the country in which the dcterms:Location occurs. http://rs.tdwg.org/dwc/terms/countryCode
locality	The specific description of the place. http://rs.tdwg.org/dwc/terms/locality
decimalLatitude	The geographic latitude (in decimal degrees, using the spatial reference system given in dwc:geodeticDatum) of the geographic center of a dcterms:Location. Positive values are north of the Equator, negative values are south of it. Legal values lie between -90 and 90, inclusive. http://rs.tdwg.org/dwc/terms/decimalLatitude
decimalLongitude	The geographic longitude (in decimal degrees, using the spatial reference system given in dwc:geodeticDatum) of the geographic center of a dcterms:Location. Positive values are east of the Greenwich Meridian, negative values are west of it. Legal values lie between -180 and 180, inclusive. http://rs.tdwg.org/dwc/terms/decimalLongitude
month	The integer month in which the dwc:Event occurred. http://rs.tdwg.org/dwc/terms/month
year	The four-digit year in which the dwc:Event occurred, according to the Common Era Calendar. http://rs.tdwg.org/dwc/terms/year
verbatimIdentification	A string representing the taxonomic identification as it appeared in the original record. http://rs.tdwg.org/dwc/terms/verbatimIdentification
phylum	The full scientific name of the phylum or division in which the dwc:Taxon is classified.http://rs.tdwg.org/dwc/terms/phylum
subphylum	The full scientific name of the subphylum in which the dwc:Taxon is classified. http://aims.fao.org/aos/agrovoc/c_331205
class	The full scientific name of the class in which the dwc:Taxon is classified. http://rs.tdwg.org/dwc/terms/class
subclass	The full scientific name of the subclass in which the dwc:Taxon is classified. http://aims.fao.org/aos/agrovoc/c_331206
order	The full scientific name of the order in which the dwc:Taxon is classified. http://rs.tdwg.org/dwc/terms/order
suborder	The full scientific name of the suborder in which the dwc:Taxon is classified. http://aims.fao.org/aos/agrovoc/c_331165
family	The full scientific name of the family in which the dwc:Taxon is classified. http://rs.tdwg.org/dwc/terms/family
subfamily	The full scientific name of the subfamily in which the dwc:Taxon is classified.http://rs.tdwg.org/dwc/terms/subfamily
genus	The full scientific name of the genus in which the dwc:Taxon is classified. http://rs.tdwg.org/dwc/terms/genus
specificEpithet	The name of the first or species epithet of the dwc:scientificName. http://rs.tdwg.org/dwc/terms/specificEpithet
infraSpecificEpithet	The name of the lowest or terminal infraspecific epithet of the dwc:scientificName, excluding any rank designation. http://rs.tdwg.org/dwc/terms/infraspecificEpithet
acceptedNameUsage	The full name, with authorship and date information if known, of the currently valid (zoological) or accepted (botanical) dwc:Taxon. http://rs.tdwg.org/dwc/terms/acceptedNameUsage
measurementRemarks	Comments or notes accompanying the measurements. http://rs.tdwg.org/dwc/terms/measurementRemarks
shape	The approximate shape of an organism. https://kos.lifewatch.eu/thesauri/traits/c_d8b26f7b
taxonRemark	Comments or notes about the taxon or name. http://rs.tdwg.org/dwc/terms/taxonRemarks
cellLength	The total distance from point to point along the longest axis of a cell. https://kos.lifewatch.eu/thesauri/traits/c_2453c25b. Cell length is expressed in micrometers (µm).
cellWidth	The shortest linear dimension of a cell, measured perpendicular to its length, from end to end along the shortest axis. https://kos.lifewatch.eu/thesauri/traits/c_3b8b8532. Cell width is expressed in micrometers (µm).
linearDimension (a,b,c,d,h)	a: The linear dimension corresponding to the distance between two points (PATO:0000122). It is usually indicated by the alphabetical code "a"; and/or "l". b: The linear dimension corresponding to the distance from one side of an object to the opposite side (PATO:0000921). It is usually indicated by the alphabetical code "b" or "d". c: The linear dimension measured through an object as opposed to its length or width (PATO:0000915). It is usually indicated by the alphabetical code "c" or "h". d: The linear dimension corresponding to the distance from one side of an object to the opposite side (PATO:0000921). It is usually indicated by the alphabetical code "b" or "d". h: The linear dimension measured through an object as opposed to its length or width (PATO:0000915). It is usually indicated by the alphabetical code "c" or "h".
density	The number of organisms, designated by taxonomic names (e.g. species, genus, family), per unit area or per unit volume. https://kos.lifewatch.eu/thesauri/traits/c_ddc0028f. Density is expressed in cells per liter (cells L⁻¹).
biovolume	Volume of an organism (cell or colony). It is calculated according to the geometric equations, after association of geometric shape to the real shape of the organism and measurements of linear dimensions (length, width). It is used for samples that are analyzed by imaging systems. https://kos.lifewatch.eu/thesauri/traits/c_117cb3e2. Biovolume is expressed in cubic micrometers (µm³).
carbonContent	Carbon content determined directly as particulate organic carbon of individual organisms or bulk communities, or indirectly by converting biovolume to carbon using empirical or theoretically derived equations. https://kos.lifewatch.eu/thesauri/traits/c_3251199b. Carbon content is expressed in picograms of carbon per cell (pg C cell⁻¹).
biomass	The amount of living organic matter per unit area or per unit volume. It can be expressed in terms of biovolume, weight or carbon content and can be measured at different organisational levels. https://kos.lifewatch.eu/thesauri/traits/c_331cd569. Biomass is expressed in nanograms (ng).
eventDate	The date-time or interval during which a dwc:Event occurred. For occurrences, this is the date-time when the dwc:Event was recorded. Not suitable for a time in a geological context. http://rs.tdwg.org/dwc/terms/eventDate
scientificName	The full scientific name, with authorship and date information if known. When forming part of a dwc:Identification, this should be the name in lowest level taxonomic rank that can be determined. This term should not contain identification qualifications, which should instead be supplied in the dwc:identificationQualifier term. http://rs.tdwg.org/dwc/terms/scientificName
scientificNameAuthorship	The authorship information for the dwc:scientificName formatted according to the conventions of the applicable dwc:nomenclaturalCode. http://rs.tdwg.org/dwc/terms/scientificNameAuthorship

### Data set 2.

#### Data set name

AbioticData_Mediterranean_BlackSea_CoastalLagoons_v1.2.csv

#### Data format

UTF-8 .csv (comma_separated values format).

#### Download URL

https://data.lifewatchitaly.eu/bitstreams/f9d327dc-b621-47c0-972b-80a284af66d0/download

#### Data format version

1.2

#### Description

The abiotic dataset includes the main physiographic, hydrological, physico-chemical, and anthropogenic pressure characteristics of the studied Mediterranean coastal lagoons.

Table 2. Abiotic data table with the terminologies used as column headers, their definitions and URIs (Uniform Resource Identifiers), based on the adopted controlled vocabularies used.

**Data set 2.** 

Column label	Column description
catalogNumber	An identifier (preferably unique) for the record within the data set or collection. http://rs.tdwg.org/dwc/terms/catalogNumber
country	The name of the country or major administrative unit in which the dcterms:Location occurs. http://rs.tdwg.org/dwc/terms/country
locality	The specific description of the place. http://rs.tdwg.org/dwc/terms/locality
month	The integer month in which the dwc:Event occurred. http://rs.tdwg.org/dwc/terms/month
year	The four-digit year in which the dwc:Event occurred, according to the Common Era Calendar. http://rs.tdwg.org/dwc/terms/year
parentEventID	An identifier for the broader dwc:Event that groups this and potentially other dwc:Events. http://rs.tdwg.org/dwc/terms/parentEventID
decimalLatitude	The geographic latitude (in decimal degrees, using the spatial reference system given in dwc:geodeticDatum) of the geographic center of a dcterms:Location. Positive values are north of the Equator, negative values are south of it. Legal values lie between -90 and 90, inclusive. http://rs.tdwg.org/dwc/terms/decimalLatitude
decimalLongitude	The geographic longitude (in decimal degrees, using the spatial reference system given in dwc:geodeticDatum) of the geographic center of a dcterms:Location. Positive values are east of the Greenwich Meridian, negative values are west of it. Legal values lie between -180 and 180, inclusive. http://rs.tdwg.org/dwc/terms/decimalLongitude
anthropogenicDisturbance	Disturbance brought about by human activity. https://w3id.org/inbio#_000092
ramsarSite	A wetland protected by the terms of the Ramsar Convention (The Convention on Wetlands of International Importance, especially as Waterfowl Habitat).http://purl.obolibrary.org/obo/ENVO_00000365
natura2000Site	Natura 2000 sites are a European network of protected areas established to conserve rare or threatened habitats and wildlife species.
localProtectionPlan	Local protection plan indicates the presence or absence of a local protection plan within the investigated ecosystem.
area	The extent of a 2-dimensional surface enclosed within a boundary. http://vocabs.lter-europe.net/EnvThes/21533 In this dataset, area represents the lagoon area, expressed in squared kilometers (km²).
outletLength	The linear extent in space from one end of something to the other end, or the extent of something from beginning to end. http://purl.obolibrary.org/obo/NCIT_C25334. Outlet length is expressed in kilometers, (km).
outletWidth	The linear extent in space from one end of something to the other end, or the extent of something from beginning to end. http://purl.obolibrary.org/obo/NCIT_C25345. Outlet width is expressed in kilometers, (km).
sinuosityIndex	Sinuosity index is a dimensionless geomorphic metric that quantifies the curvature of rivers, streams, and lagoons. It is commonly used to classify transitional water ecosystems, as channel curvature influences hydrodynamics and, consequently, the abundance and distribution of benthic macroinvertebrates and phytoplankton guilds.
tidalRange	The difference in height between the high tide and the low tide. https://kos.lifewatch.eu/thesauri/sandybeachthes/c_a0ef4653. Tidal range is expressed in meters, (m).
waterBodyAmmonium	Concentration of ammonium per unit volume of a river, estuary, sea or ocean by any method in filtered or unfiltered water. http://vocab.nerc.ac.uk/collection/P35/current/EPC00009. Ammonium concentration is expressed in micromoles per liter (µmol L⁻¹).
nitrite	Concentration of nitrite nitrogen per unit volume of the water column. https://kos.lifewatch.eu/thesauri/vrephytoplankton/c_813004bb. Nitrite is expressed in micromoles per liter (µmol L⁻¹).
nitrate	Concentration of nitrate nitrogen per unit volume of the water column. https://kos.lifewatch.eu/thesauri/vrephytoplankton/c_7249c816. Nitrate is expressed in micromoles per liter (µmol L⁻¹).
dissolvedInorganicNitrogen	The sum of nitrate, nitrite, and ammonium concentrations. http://vocabs.lter-europe.net/EnvThes/20833 Dissolved inorganic nitrogen is expressed in micromoles per liter (µmol L⁻¹).
phosphate	Phosphate concentration in the water sample. https://vocabulary.actris.nilu.no/actris_vocab/phosphate. Phosphate is expressed in micromoles per liter (µmol L⁻¹).
silicate	Concentrations of dissolved inorganic silicon usually as hydrosilicic acid (often called silicate) and biogenic silica. http://vocab.nerc.ac.uk/collection/P36/current/SLCATE/ Silicate is expressed in micromoles per liter (µmol L⁻¹).
depth	Depth is the vertical distance below the surface. http://vocab.nerc.ac.uk/collection/P07/current/CFSN0721/. Depth is expressed in centimeter (cm).
waterTemperature	Water temperature measured in situ. https://kos.lifewatch.eu/thesauri/vrephytoplankton/c_a343112a. Water temperature is expressed in Celsius degree (°C).
conductivity	The ability of an object to conduct an electric current and resistivity is a physical quantity that reflects the electrical conductivity of a material. http://vocab.nerc.ac.uk/collection/P14/current/GVAR0165/. Conductivity is expressed in microsiemens per centimeter (µS cm⁻¹).
totalDissolvedSolids	Total concentration of dissolved substances (such as calcium, magnesium, sodium, chlorides, sulfates, and bicarbonates) and small amounts of dissolved organic matter in water. https://kos.lifewatch.eu/thesauri/vrephytoplankton/c_2b1486ad. Total dissolved solids are expressed in micrograms per litre (µg L⁻¹).
salinity	Salinity is the saltiness or dissolved salt content of a body of water (see also soil salinity). Salinity is an important factor in determining many aspects of the chemistry of natural waters and of biological processes within it, and is a thermodynamic state variable that, along with temperature and pressure, governs physical characteristics like the density and heat capacity of the water. http://vocabs.lter-europe.net/EnvThes/10312 Salinity is expressed in Practical Salinity Units (PSU).
oxygenSaturation	Oxygen saturation in water measures dissolved oxygen (DO) relative to the maximum possible concentration, expressed in percentage (%). http://vocabs.lter-europe.net/EnvThes/21458
dissolvedOxygen	Dissolved oxygen (DO) is the amount of oxygen that is present in water. http://vocabs.lter-europe.net/EnvThes/20851. Dissolved oxygen is expressed in milligrams per liter (mg L⁻¹).
pH	Minus the log of the hydrogen ion concentration (moles per litre) in the water column. http://vocab.nerc.ac.uk/collection/P09/current/PHPH/
eventDate	The date-time or interval during which a dwc:Event occurred. For occurrences, this is the date-time when the dwc:Event was recorded. Not suitable for a time in a geological context.http://rs.tdwg.org/dwc/terms/eventDate

## Figures and Tables

**Figure 1.**
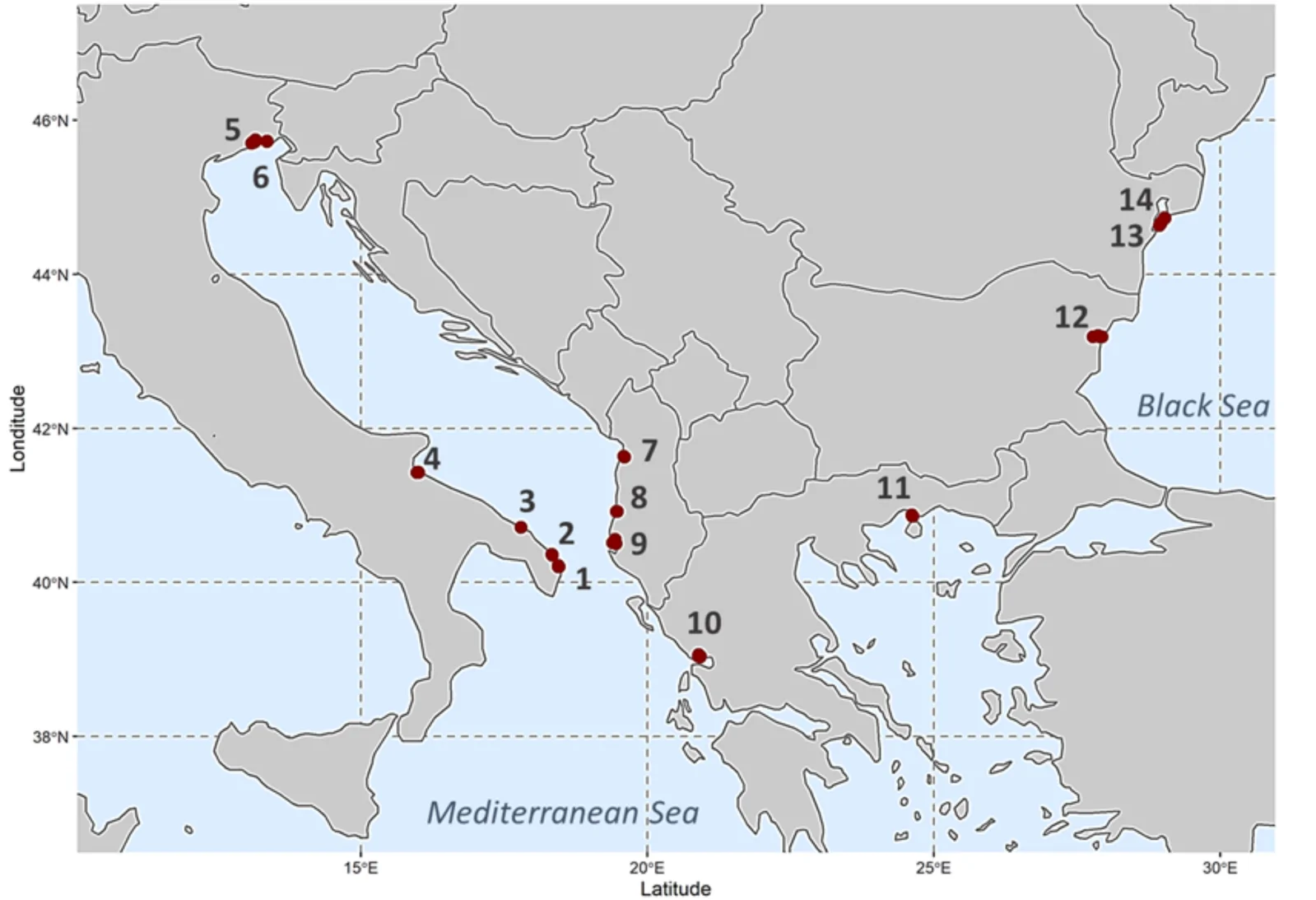
Map of Mediterranean and Black Sea sites included in the dataset. The 14 sites are 1-Alimini, 2-Cesine, 3-Torre Guaceto, 4-Margherita di Savoia, 5-Grado Valli da Pesca, 6-Grado Marano, 7-Patok, 8-Karavasta, 9-Narta, 10-Logarou, 11-Agiasma, 12-Varna Bay, 13-Sinoe lagoon and 14-Lehaova.

**Figure 2.**
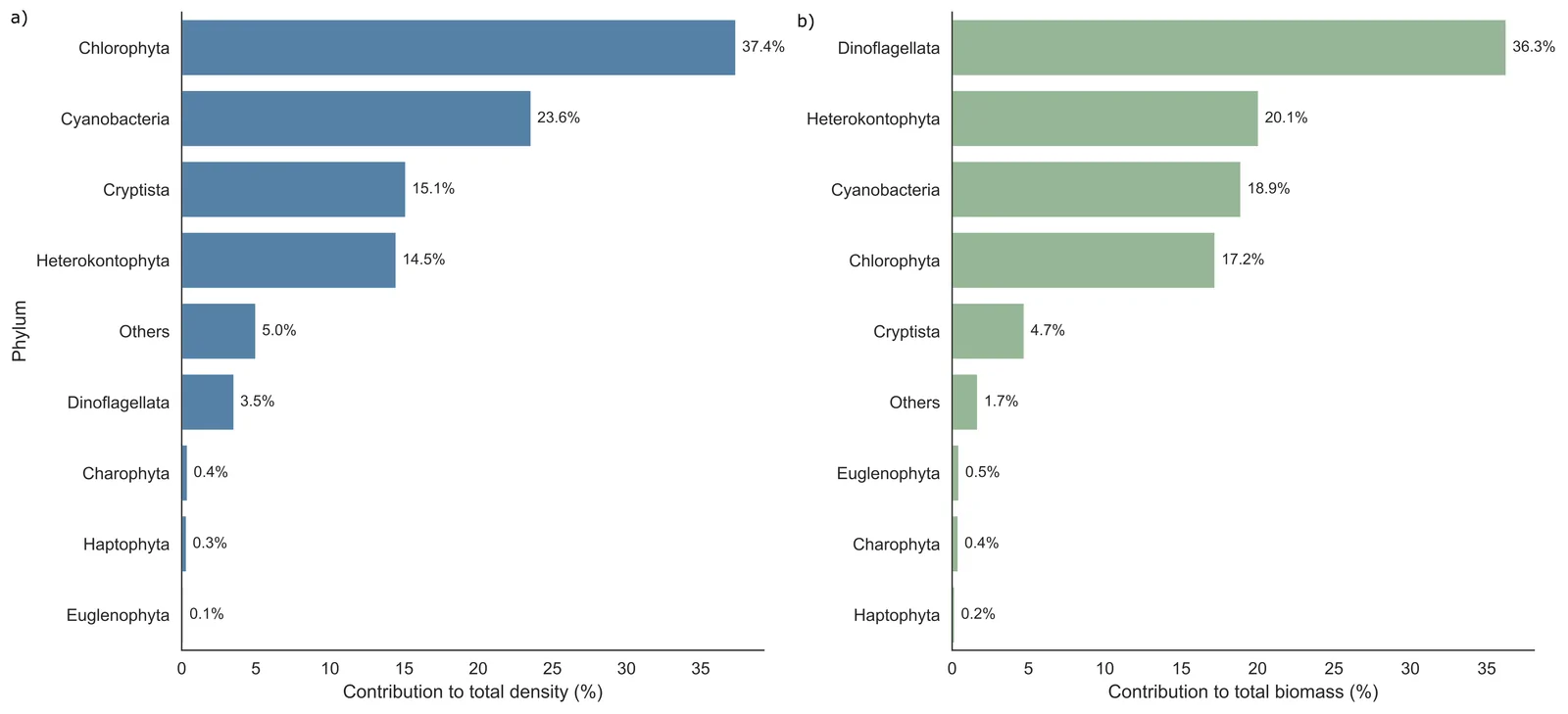
Taxonomic composition of the phytoplankton dataset aggregated at phylum level. **a** Relative contribution based on total density; **b** Relative contribution based on total biomass.

**Figure 3.**
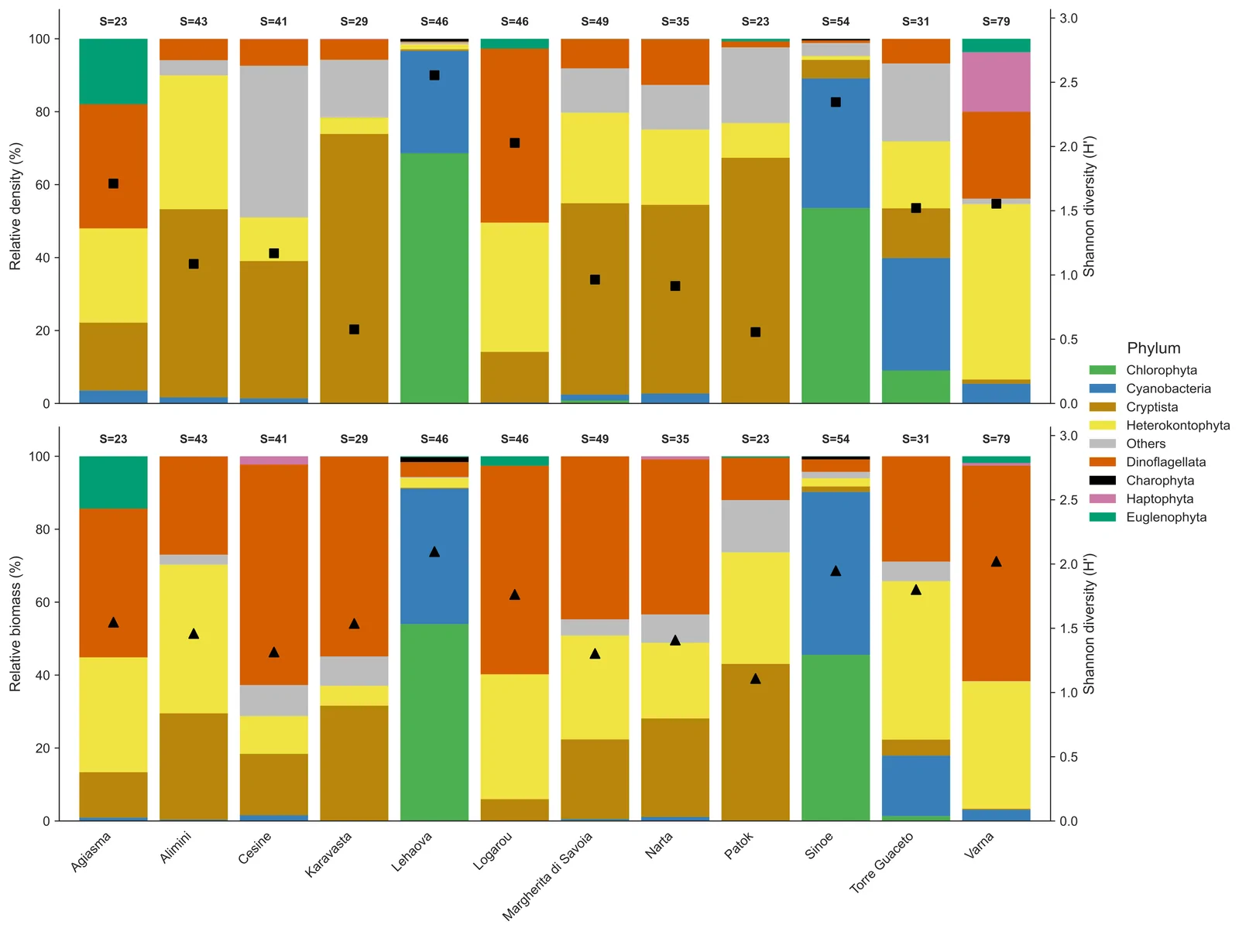
Phytoplankton composition across the studied lagoons. Stacked bars show the relative contribution (%) of major phytoplankton phyla based on density (top) and total biomass (bottom). Values represent the mean composition across sampling events within each locality. Taxonomic richness (number of taxa) is indicated by S, and black symbols represent Shannon diversity (H') calculated from density (squares) and biomass (triangles).

**Figure 4.**
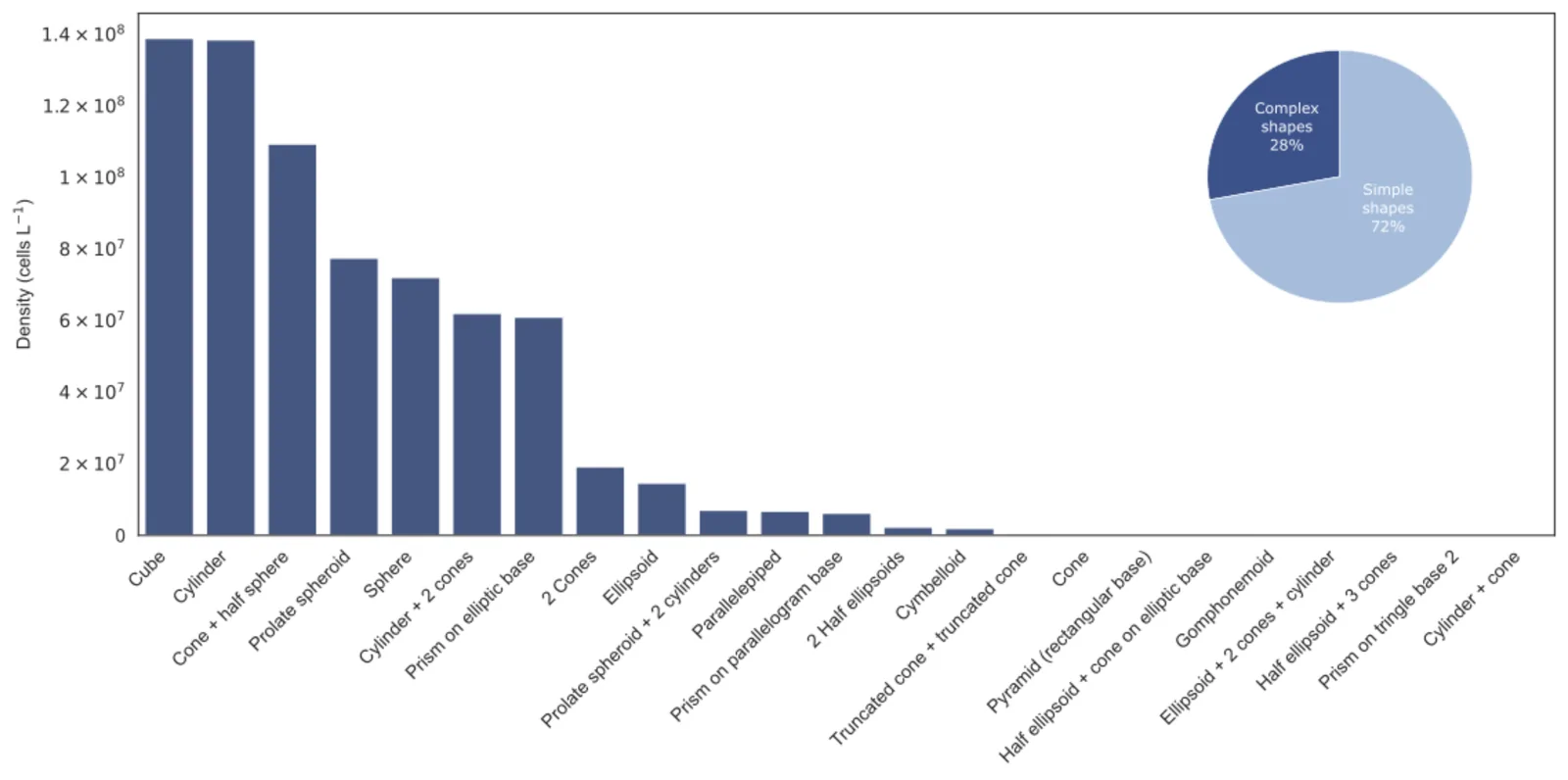
Shape distribution of phytoplankton cells and relative contribution (in %) of simple and complex shapes in terms of cell density.
